# Women’s perceived working conditions in the mining industry: A qualitative study

**DOI:** 10.4102/hsag.v28i0.2212

**Published:** 2023-12-14

**Authors:** Masesi M. Mahlasela, Mankuku M. Madumo, Moreoagae B. Randa

**Affiliations:** 1 Department of Nursing Science, School of Health Care Sciences, Sefako Makgatho Health Sciences University, Pretoria, South Africa; 2 Department of Public Health, Sefako Makgatho Health Sciences University, Pretoria, South Africa

**Keywords:** industry, mining, perceptions, women, workplace, working conditions

## Abstract

**Background:**

Women’s status in society has been contested over the years, with arguments centred on the deliberate marginalisation of women by ancient policies and legislations, which compelled women to assume secondary status in society.

**Aim:**

This study aimed at exploring and describing the perceptions of women on working conditions in the mining industry. A qualitative, explorative and descriptive design was followed.

**Setting:**

The study was conducted at a mining industry based in Mpumalanga Province.

**Methods:**

Ten women were purposively selected to participate in the study. Data were collected through in-depth semi-structured face-to-face interviews. Data were analysed using Tech’s method of qualitative analysis.

**Results:**

Three themes and categories emerged from data analysis. The themes that emerged were: Benefits for women in the mining industry, work conditions-related challenges for women in the mining industry and opportunities for growth and development of women in the mining industry.

**Conclusion:**

Despite the employment of women in the mining industry, the women echoed that mining remains a male-dominated place of employment.

**Contribution:**

The study revealed that although the mining industry is still a male-dominated environment, women are generally content to be working at the mines. A lot of transformation should take place to make mining a women-friendly place of employment. The relevance of this study for mental health is that gender equality is a risk factor for gender-based violence. Furthermore, facing discrimination can also result in anxiety and psychological trauma that can negatively affect a woman’s sense of well-being and success.

## Introduction

Globally, the mining industry has been dominated by the perception that it was exclusively for male workers (De Klerk 2012). This perception was even enforced by legislation set out by various governments. In the early 1900s, Article 2 of the International Labour Organization’s (ILO) *Convention 45 of 1935* was implemented, forbidding the inclusion of women in underground mining. A study conducted on the status of women in Canada revealed that women were still facing major challenges in terms of employment in the mining industry (Women in Mining Canada 2010).

In South Africa, mining as an occupation was traditionally reserved for men only, and the mining law prohibited women from underground operations employment (Mangaroo-Pillay & Botha 2020). The authors, Botha and Cronjé (2015) shared the consensus that the exclusive reservation of the mining industry for men is based on the nature of core mining jobs that require physical strength and are associated with high risk. Cultural perspectives also prevented women from working in surface mines or in surface occupations (Moyo 2011). In 2001, the *South African Minerals Act 50 of 1991* forbade women to be employed in the mining industry. The *Mineral and Petroleum Resources Development Act 28 of 2002* then replaced the Act. *The Mine Health and Safety Act 29 of 1996* and the Broad-based Socio-economic Empowerment Charter (signed in 2002 and published in 2004) were introduced to address the imbalances in the industry, calling for 10% of the workforce to be women by 2009 (South Africa 2002, 2004).

These two acts opened channels for women in the mining industry where the issue of diversity was addressed. According to these acts, everyone irrespective of gender could work at places of their choice, even in the mining industry (Benya 2009). Since legislation allowed women employment underground, mining has made a significant change in gender equity. According to Coetzee and Schreuder (2010), in South Africa, there has been an increase of women in top management positions in mining from 0.01% to 9.3%. Senior management positions for women in mining in South Africa have increased from 0.03% to 10.01% (Coetzee & Schreuder 2010). This was, however, a new phenomenon as the mining industry had been regarded as a male-dominated environment for a long time. Accepting women underground has not been easy for mining companies and has led to many difficulties in meeting the needs of women in the mining industry.

Hancock (2014) states that 11% of the total workforce in the South African mining industry is comprised of women. However, the exact percentage of those in engineering and geology cannot be determined, and this accounts for under representation in the South African mines. There has also been a significant increase in women in middle management and professional positions from 5.4% to 83% in the mining industry in South Africa (Coetzee & Schreuder 2010).

Despite the increase in the number of women in senior positions in the mining industry, women still feel that somehow they are not equally treated like their fellow male colleagues (Coetzee & Schreuder 2010). Several authors who conducted research on women employed in South African mines since the inception of the *Mineral and Petroleum Resources Development Act 28* in 2002 have revealed that the employment of women remains a challenge and that women still face barriers such as unequal treatment of men and women to some extent (Botha 2016, 2017; Chamber of Mines of South Africa 2017; Mavuso 2015; Ntombela 2014).

The trade unions in South African mines have shown to be unsupportive of women in this regard. They still have the negative perception that women are not suited for the mining industry. Trade unions are hostile towards women and as such do not accommodate women issues in their meetings (Mlambo 2011). In cases where women have skills, they are said to be lacking the ability to perform some tasks like drilling or pipe installation because of their physical build. They are given lighter jobs, and this has a negative impact on their career paths; therefore, they miss promotional opportunities (Benya 2009). Although great efforts have been made to employ women in the mining industry, women working in core mining areas are often seen as sexual objects and experience physical, verbal and non-verbal abuse (Botha 2016; Creamer Media 2019; Nene 2016). Considerable progress has been made in accommodating women by providing personal protective equipment (e.g. overalls, dust masks and safety boots) designed for women, but improvements are still needed in this regard. Furthermore, a need for protective clothing for pregnant women has been identified (Botha 2017; Mine Health and Safety Council 2015).

Research conducted on women employed in South African mines since the inception of the *Mineral and Petroleum Resources Development Act, No. 28 of 2002* (MPRDA) has revealed that the employment of women remains a challenge and that women still face barriers to some extent (Chamber of Mines of South Africa 2017; Hancock 2014). Since the introduction of the South African Mining Charter, there have been an estimated 600 000 women in the mining industry (office personnel and cleaners included in the Southern African Development Community [SADC] region) (Mlambo 2011). Literature reveals that despite the increasing number of women employed in the mining industry, the equal treatment for men and women remains unguaranteed. The trade unions in South African mines have shown to be unsupportive to women. They still have the negative perception that women are not suited for mine work. Trade unions are hostile towards women and as such do not accommodate women issues in their meetings (Mlambo 2011).

## Problem statement

Literature indicates that women mineworkers in South African mines found that whilst verbal harassment is most common, face requests for sexual favours in exchange for physical labour or for promotions, transfers or changes in work schedules with verbal harassment being the most common (Keetharuth 2021). Most incidents of abuse were seldom reported, as the abused women feared losing employment, while some women did not report the abuse because they did not know their rights. However, in other cases, the abuse was reported but it was never dealt with or followed up as the most senior person (Rawoot 2014). Since legislation allowed women employment underground, mining has made a significant change in gender equity. However, women still feel that somehow they are not equally treated like their fellow male colleagues (Coetzee & Schreuder 2010). According to Phiri (2021), there are socio-economic, cultural and legal complexities associated with the participation of women in the mining industry in South Africa, which are not well understood to guide appropriate policies and actions to enhance women role in mining. It is this knowledge gap that the study seeks to make a contribution by investigating the role of women in the mining industry in Chaneng, Rustenburg. The findings of this study would reveal work-related challenges faced by women in this mining industry and in making the mining environment conducive for women to work in.

## The purpose of the study

The purpose of this study was to explore and describe the perceptions of women miners regarding their working conditions in the mining industry in Mpumalanga Province.

## Methods

A qualitative, explorative and descriptive research design was employed to explore and describe the perceptions of women on their working conditions in the mining industry. A non-probability purposive sampling procedure was followed to select the participants who were knowledgeable and experienced on the phenomenon under investigation (Brink, Van der Walt & Van Rensburg 2016). In-depth, face-to-face individuals’ interviews using semi-structured questions were used to collect data. According to Polit and Beck (2017), face-to-face interviews have high response rates, as participants are less likely to refuse to talk to an interviewer or to ignore a question. Face-to-face interviews allowed participants an opportunity to give their answers in their own words and to express their own opinions.

### Research setting

South Africa is a country that is rich in several mineral resources ranging from gold to coal, vanadiferous magnetite, cobalt, iron, copper and others. Mpumalanga is a province that is rich in mineral resources. It has a total of seven mines that manufacture coal, silica and excavate, refine and transport minerals. The research setting was limited to mining industry based at Secunda in Mpumalanga Province. The research setting was limited to mining industry based at Secunda in Mpumalanga Province. Secunda, is a modern company town (built after 1974). It is located about 80 miles (130 km) east of Johannesburg in a region of extensive coal reserves and adequate water supplies. The industry deals with the sifting, cleansing, testing, preparing, importing and exporting of coal. The mine was selected by the researcher on convenient sampling, as it was the unit where most women were employed. Categories of workers ranged from senior management to engineers, artisans, safety officers, environmental officers, clerks and general workers. Both men and women share the same jobs.

### Population

The target population for this study consisted of women employed at Mpumalanga mine, who were available at the time of data collection. Non-probability convenience sampling was used, as the participants were available by virtue of being accessible to the researcher (Bryman et al. 2014). The total population comprised 460 employees, of whom 96 were women. The study sample consisted of a segment of 12 women who were working in this section and met specific characteristics that were of interest to the researcher.

The study population was comprised of women miners who participated in the interviews. The study was based on the feminist research theory, which informs the interconnections among the controversies of heterogeneity enveloping social class, race and gender and to bring forward the interests, health and security of women in all constituents of the world (De Vault & Gross 2012; Hesse-Biber 2012). The feminist theory was appropriate for this study as it highlighted gender-based disparities and sought new ways of constructing knowledge and improving practice.

### Participants’ recruitment and sampling

The participants were recruited during information sharing sessions. The sessions included the daily safety meetings held every morning at the beginning of every shift, and the researcher would give health education on health issues as an Occupational Health Nurse (OHN). Communication sessions were held every Wednesday and health education sessions shared among the safety, health, hygiene and environmental departments. The researcher had no relationship with the participants. The participants were from both surface and underground sections of the mine, while the researcher was an OHN. It was important for the researcher to choose the participants based on judgement as the study did not aim to generalise the conclusions from the collected data (Polit & Beck 2017). A non-probability purposive sampling was used to select the participants based on the knowledge they had about the experiences of working conditions within the mining industry. The selection criteria included women of all races who were willing to participate in the study. In addition, women workers both in permanent posts and on contract were included and they needed to have at least 6 months’ employment experience in the mining industry. It is worth noting that all participants who were employed at the time of data collection were above 24 years of age.

### Data collection procedure

#### Research instrument

The research study employed in-depth, face-to-face individual interviews using semi-structured questions to extract important data that informed the study (Ehrlich & Joubert 2014). To test for efficacy, the data collection tool was pre-tested on three women who met the inclusion criteria of the participants needed for the study. No modifications were made on the tool based on the results.

#### Data collection

The face-to-face individual interviews were appropriate as they have a high response rate as well as allowing for additional information as the interviewer observes the participants’ behaviour, which can assist in interpreting responses of participants (Polit & Beck 2017). In addition, the researcher can follow up on the most interesting points with this method, enhancing collection of useful and rich data quickly.

After ethical permission was granted, the researcher sought permission from the research site and the participants after the study objectives were explained to ensure understanding. The data was collected by the researcher, a professional nurse who attended a course in communication skills and also completed a research methodology module in a recognised institution.

The interviews were conducted in the boardroom, which was noise-free and a familiar place to the participants as they used the area to hold their meetings and information sessions. A suitable arrangement of a table and a chair was made. A ‘Please do not disturb, interviews in progress’ sign was put on the door. Each interview session lasted for approximately 30 min to 1 h. Individual appointments were made according to dates and times that were convenient for the participants. The face-to-face interviews were conducted in February to March 2019.

A structured interview guide with open-ended questions was used to gather in-depth information, and this was supported with field notes and the use of an audio recorder (Doyle et al. 2019). The field notes were taken during the interviews and included observation and theoretical notes.

The languages used to conduct the interviews were IsiZulu and English, which gave participants an opportunity to express themselves freely. The research question posed was ‘What are your perceptions on working conditions as a working woman in the mining industry?’ The responses were followed up by asking probing questions throughout the interview process. Bracketing was done through identifying and laying aside own opinions and preconceptions concerning the study (Polit & Beck 2014). Data collection continued until saturation was reached at participant number 10. Data saturation was noted when data became redundant and there was no emergence of new data from the participants (Ravitch & Carl 2019).

### Data analysis

Data were analysed manually using a thematic method described by Tesch (De Vos et al. 2011). Data collection and data analysis proceeded concurrently. The observed non-verbal cues were documented and interpreted by the researcher, while the theoretical notes were transcribed verbatim and utilised in preliminary data analysis.

The researcher carefully read the transcripts and grouped similar themes together according to similarities. Coding of qualitative data, content analysis and development of themes were done (Creswell 2014). The service of an independent coder who is experienced in qualitative data analysis methods was requested. A consensus meeting was then held between the researcher and the independent coder to finalise the themes, categories and sub-categories that emerged from the data.

### Trustworthiness

The principles of credibility, dependability, transferability and authenticity were applied to enhance trustworthiness. The researcher made sure that the women understood the research question, by rephrasing the questions where they seemed unclear to the participant. Participants were also informed about the study purpose and the processes before they signed the informant consent. Emphasis was also made that they can withdraw from the study at any time without stating the reason and no penalty will be incurred. To enhance confirmability, clarification of participants’ responses was done to ensure that the perceptions were those of the participants and not those of the researcher. Enlisting an independent coder during data analysis also ensured peer debriefing.

An audit trail, audio-recorded and transcribed data as well as verbatim quotes from participants were used to ensure dependability. Descriptive data was provided to enable other researchers to evaluate the applicability of the study to other contexts. Authenticity was ensured using verbatim quotes from participants’ interviews.

### Ethical considerations

Approval to conduct the study was granted by the Ethical Committee of Sefako Makgatho Health Sciences University before data collection (SMUREC/H/123/2017: PG).

To ensure the ethical compliance of this study, permission was also sought from the Vice-President of the Mining Industry in Mpumalanga, the Vice-President, Safety, Health and Environment and the Legal Department of Mining in Mpumalanga.

Informed consent as well as permission for audio recording was sought from the participants before data collection commenced to ascertain that they were well informed about the purpose and the process to be followed regarding the study. It was emphasised that participation was voluntary, and the women were informed that they could withdraw from the study at any time without being penalised and providing the reasons.

## Results

The study sample consisted of 10 women miners who voluntarily participated in the face-to-face semi-structured individual interviews (Table 1). The participants’ profile included age, race, gender, level of education, job levels and years of employment. The sample comprised of both black and white women mineworkers with the majority being black. The women’s years of experience varied with half of the women being with the industry for a period of 5 years to 9 years and two of the women having 15–24 years of employment within the industry. Two of the women were in management positions while the other eight occupied skilled and semi-skilled positions.

**TABLE 1:** The biographic data of participants.

Category	Number
**Age (years)**	
25–34	5
35–44	3
45–54	2
**Race**	
Black people	8
White people	2
**Gender**	
Women	10
**Level of education**	
Grade 12	5
Technical certificate	3
Diploma or degree	2
**Job levels**	
Management position	2
Skilled labour	3
Semi-skilled labour	5
**Years of employment**	
5–9	5
10–14	3
15–24	2

## Discussion of themes

The key themes that emerged from the analysed data relate to perceptions of women regarding their working conditions in a mining industry, opportunities for growth and development for women. The summary is indicated in Table 2.

**TABLE 2:** Summary of the themes and sub-themes.

Themes		Sub-themes
1	Benefits for women	Medical aid Leave allocation
2.	Work conditions related challenges for woman in mining industry	Male harassment
		Remuneration
		Promotions
		Work distribution
3.	Opportunities for growth and development of woman	Learnerships

### Theme 1: Benefits for women

The mining company provided several benefits, and the women participants were satisfied with those.

#### Medical Aid

The medical aid benefit was made available for every employee irrespective of his or her gender, and no external medical aid was allowed for the industry’s employees:

‘The medical aid is very expensive and compulsory but very good though. Nobody can have an external medical aid. Only the in-house medical aid is allowed.’ (Participant 3, female, 36 years old)

‘Okay. I would say, we are getting the same benefits. I am thinking about benefits such as medical aid. Everyone gets medical aid during the employment with the industry.’ (Participant 9, female, 39 years old)

#### Leave allocation

In terms of leave, the participants indicated that varying categories of leave are available to all employees. Such categories included maternity, annual, family responsibility and sick leave. Participants were all satisfied with the maternity leave benefit as enough time to care for the baby after delivery was provided:

‘As for the maternity leave, we get six months which gives us enough time to bond with our babies before we can start normal duties. I am satisfied with that; it is very nice.’ (Participant 8, female, 45 years old)

The women shared that they were entitled and encouraged to take leave and have time away from work to avoid fatigue. Furthermore, they were allocated equal number of days for family responsibility and compassionate leave:

‘Some people like selling their leave days and don’t want to take leave, the company will force you take the 12 consecutive days leave so that we are not tired. This is a very good thing.’ (Participant 5, female, 31 years old)

‘All employees get the same number of days for the family responsibility and compassionate leave. We all get four days. It is given to you every year, but it does not accumulate. If you do not take it, you forfeit it.’ (Participant 4, female, 33 years old)

Furthermore, the participants were satisfied with the sick leave policy in place, and they shared that the company made sure that employees do not abuse the sick leave and that strict measures were in place:

‘There is sick leave for every employee. I think it is 90 days for three years. I know I have never used it, but it is there. People started misusing sick leave then the company introduced strict laws. You cannot be sick for five days and be at home, at least you must be admitted, and then the company does not complain.’ (Participant 8, female, 45 years old)

### Theme 2: Work conditions related challenges for women in mining industry

Data from the interviews provided evidence that a lot still needs to be done in terms of gender equity, career development and progression of women in the mining industry. The following were some of the concerns raised:

#### Male harassment

The participants verbalised that they experienced both verbal and sexual harassment by male colleagues. Lewd comments were passed by men about the women’s body parts as well as being inappropriately fondled and these actions made them feel disrespected. Although the industry had policies against sexual harassment, participants were not sure about the implementation thereof. Previously reported cases were not acted upon, and this discouraged the participants from reporting. Fear of victimisation, dismissal, or non-promotion after reporting the harassment was also a concern raised by some participants:

‘There was a case whereby my supervisor touched the bums of a female, but in that case, that supervisor won the case. After that, the female was moved to another section. It happened, the man will spank your bum or make funny comments about your bum that would be a form of sexual harassment, but nothing will be done to him even if you try to report him.’ (Participant 10, female, 34 years old)

‘There are policies. I remember our HR called us to teach us about sexual harassment, but I don’t think the policies work in this environment. Sometimes the harassment is verbal, sometimes sexual.’ (Participant 4, female, 33 years old)

‘A man will touch you in front of everyone, of course! Then you tell him you don’t like what he is doing but he will keep doing it. Then you would think of taking the matter to HR, but again think about being victimized or not getting promoted. Men harass us emotionally when they refuse to help us, and they tell us that we get paid for doing the job. Yes, we get paid but there are some tasks that we cannot do alone, like picking up heavy objects.’ (Participant 1, female, 29 years old)

#### Remunerations

Although remuneration was treated as a confidential issue, the participants shared the same perception and/or feeling that men were paid higher salaries. The perception was based on the men’s luxurious lifestyle and driving of expensive cars:

‘The wages, hey, I wouldn’t know. I am not sure because your payslip is confidential. Whatever you are getting is confidential. So, I wouldn’t know, I would be lying if I said I know who’s getting more between men and women.’ (Participant 6, female, 44 years old)

‘If you listen to what the people talk on the floor, they say men are getting more but we will never know because it is a policy that we don’t talk about what we are getting. So, we are not allowed to share that type of information with each other, but you can just hear from what people are saying that men are getting more than the women, in the same positions. Yes, irrespective of everything. It sounds like men are getting the higher salary than the women.’ (Participant 5, female, 31 years old)

#### Promotions

Although the benefits were equal, women felt that there is discrimination and disparity in terms of learner ship opportunities and only men occupied promotions and high-level positions. The same inequality was noted regarding available opportunities for training as men were considered over women:

‘At the place where I am working, there is no female on a higher position. I’m talking about one being a foreman, a shift boss, there is nothing like that? They don’t believe that women can also be in higher positions, especially management positions, yes.’ (Participant 10, female, 34 years old)

‘But men do get promoted faster than women, yeah. Promotions according to my view will be given to men first even if you apply for the job in time, the guy will get it before you as a woman.’ (Participant 8, female, 45 years old)

‘When learner ships open, they always take men. It is very rare to see a female learner. Even those who come to train, they are from the mines but nothing from surface. I have never seen a female learner here at the workshop!’ (Participant 1, female, 29 years old)

The participants also highlighted that women of childbearing age were not considered for promotion. This was justified, as that would put the company’s production output at risk when one goes on maternity leave:

‘They wouldn’t take the risk of promoting me, because I am still at the child- bearing age so I might fall pregnant three months after I am promoted. I remember in 2009 we were promised a crèche or a day-care centre, but it never happened! They are very good in promising!’ (Participant 2, female, 32 years old)

#### Work distribution

Unfair distribution of work was reported by women as they were allocated difficult or heavier tasks than men. Women artisan workers are allocated individually while men are allocated as a group; and this affected their output and therefore their appraisal:

‘We are given tough work and men are given simple jobs. As a female artisan, I am always given the task of pulling these heavy cables, yes, alone.’ (Participant 2, female, 32 years old)

‘No, the work is not fairly distributed. I think as women, we are out to be tested to check if we are strong to work here. Why are we always given tough work? Men do not do anything underground; all the dirtiest and difficult work is distributed to us.’ (Participant 7, female, 54 years old)

‘You lift up the heavy object, like a cable a male will never help you. The only thing he will tell you is that “my own wife is at home”. The older men are better than the younger men, at least they sometimes help but **[Yhooo]** they complain thereafter saying it is not their job to help women – they came to work for their families.’ (Participant 9, female, 39 years old)

### Theme 3: Opportunities for growth and development for women

The data collected from the participants revealed that opportunities for growth and development in the mining industry were through learner ships. However, of concern was that women were not considered when opportunities for learnership programmes became available.

#### Learnership

The women indicated that only men were granted opportunities to study and develop:

‘We as women also want to be selected for learnerships, we want to upgrade our education levels! Maybe they should investigate their selection process so that women can also be accommodated.’ (Participant 1, female, 29 years old)

‘We want to see women in higher positions but that cannot happen if women are not selected for learnerships. It depends, but I think if they can give me a chance to be a learner, I will prove myself and next time when you come here, I will be a mine captain, yeah…’ (Participant 3, female, 36 years old)

## Discussion

The aim of the study was to explore and describe the perceptions of women on working conditions in the mining industry in Secunda, Mpumalanga Province. The study results revealed that women working in the mining industry were generally satisfied with the leave benefits provided. The practice of granting employees leave was in accordance with the *Basic Conditions of Employment Act (BCEA) No: 75 of 1997*, Sections 20 and 23.

Complaints of male harassment were also reported from the findings of this study. Verbal, sexual and/or emotional abuse were the forms of harassment that were mentioned. The findings, furthermore, indicated that men used unacceptable language and comments in a form of verbal harassment. This finding lent credence to the research that was conducted by De Klerk, Botha and Botha (2015), which indicated that numerous harassment manifestations such as threats, demands and bodily contact between men and women working in the mining industry were reported by women. Additionally, Mining Safety (2013) shared that gender discrimination and sexual harassment could affect women’s psychological health, generating stress-related reactions such as emotional trauma, anxiety, depression, anger and low self-esteem and that can also affect their physical health, causing stress-related diseases such as sleep disorders, headaches, stomach problems and ulcers. Kendare (2017) in alignment with the findings of this study, indicated that harassment still existed in the mining industry in South Africa. Research conducted in various countries indicates that across the globe, women working in the core business of the mining sector are still viewed as sexual objects and are still subjected to sexual harassment of some kind (Botha 2016).

The findings also indicated the need for learnership opportunities and promotions, which favoured only men and excluded the female gender. Furthermore, the women were desperate for training and career development support for example, study leave and mentoring systems (Jones & Moalusi 2019; Matshingane 2017; Mavuso 2015; Slater 2018). These findings are also supported by the literature review that suggests that, globally, women face challenges regarding insufficient professional and career development, which include poor mentoring systems and career paths. Although women’s participation in the mining labour force has increased over the years, women’s position, development and advancement in the workplace continue to be subordinate to that of their male counterparts (Ledwaba & Nkomo 2021). Despite the availability of various equity employment opportunity legislations, there are still barriers hindering women’s advancement in the mining industry.

The mining industry must be made aware of the women’s needs in terms of training and career development. Transparent workplace opportunities should be shared, which include training and skills development opportunities, career development opportunities and financial assistance; this would not only contribute towards a skilled workforce but would also empower women to do their work effectively and could enhance productivity, personal satisfaction and job enrichment (Nel et al. 2012). The development and mainstreaming of women in the core business of mining is critical for reaching employment targets, as prescribed by the Mining Charter, as well as for retaining women for the mining industry (Botha 2014).

## Conclusion and recommendations

The study findings revealed that women workers in the mining industry still encounter challenges despite the available approved regulations and policies in place. Although the participants were content and happy with the benefits offered, gender equity was still an issue. This was evident as there was an imbalance between men and women in terms of senior positions, empowerment opportunities, promotions and work distribution. Men were given preference over their female counterparts.

Although women have become an essential part of the mining labour force, their position, development and advancement in the workplace still remain largely in the minority in decision-making positions. The women are furthermore regarded as being subordinate to their male counterparts. Despite various equal employment opportunity legislation and policies in place, many hidden barriers still exist that prohibit women from advancement and career growth in the industry. A lot still has to be done to mitigate the barriers and to ensure women’s full participation and consideration of women in the global mining labour force.

Furthermore, the study indicated that if women were treated with dignity and respect, then more women would be attracted to the industry. Recommendations included regular evaluation of the *Mine Health and Safety Act No. 29 of 1996 (MHSA)* to assess its effectiveness and if the mechanisms for addressing gender-based discrimination are implemented in the mining industry as this was found to be a common issue shared in the previously conducted research studies. Lack of opportunities for growth and development, as well as both lack of support and resources for women are the factors that can exert a negative force on the mental health of women.

### Limitations

The study was conducted only in one mining industry. Therefore, the limited number of the participants means that the findings of the study may not be generalisable to other industries and a larger population.
